# Correction: Repertoire characterization and validation of gB-specific human IgGs directly cloned from humanized mice vaccinated with dendritic cells and protected against HCMV

**DOI:** 10.1371/journal.ppat.1009385

**Published:** 2021-03-01

**Authors:** Sebastian J. Theobald, Christoph Kreer, Sahamoddin Khailaie, Agnes Bonifacius, Britta Eiz-Vesper, Constanca Figueiredo, Michael Mach, Marija Backovic, Matthias Ballmaier, Johannes Koenig, Henning Olbrich, Andreas Schneider, Valery Volk, Simon Danisch, Lutz Gieselmann, Meryem Seda Ercanoglu, Martin Messerle, Constantin von Kaisenberg, Torsten Witte, Frank Klawonn, Michael Meyer-Hermann, Florian Klein, Renata Stripecke

There is an error in the entry of the author Constantin von Kaisenberg’s name in the XML of the article, where ‘von’ is entered as a middle name. The full surname should be ‘von Kaisenberg’.
