# Supplementary figures and images for: Repertoire characterization and validation of gB-specific human IgGs directly cloned from humanized mice vaccinated with dendritic cells and protected against HCMV
Source: PLoS Pathog. 2020 Jul 15;16(7):e1008560. doi: 10.1371/journal.ppat.1008560 (PMC7363084; doi:10.1371/journal.ppat.1008560)

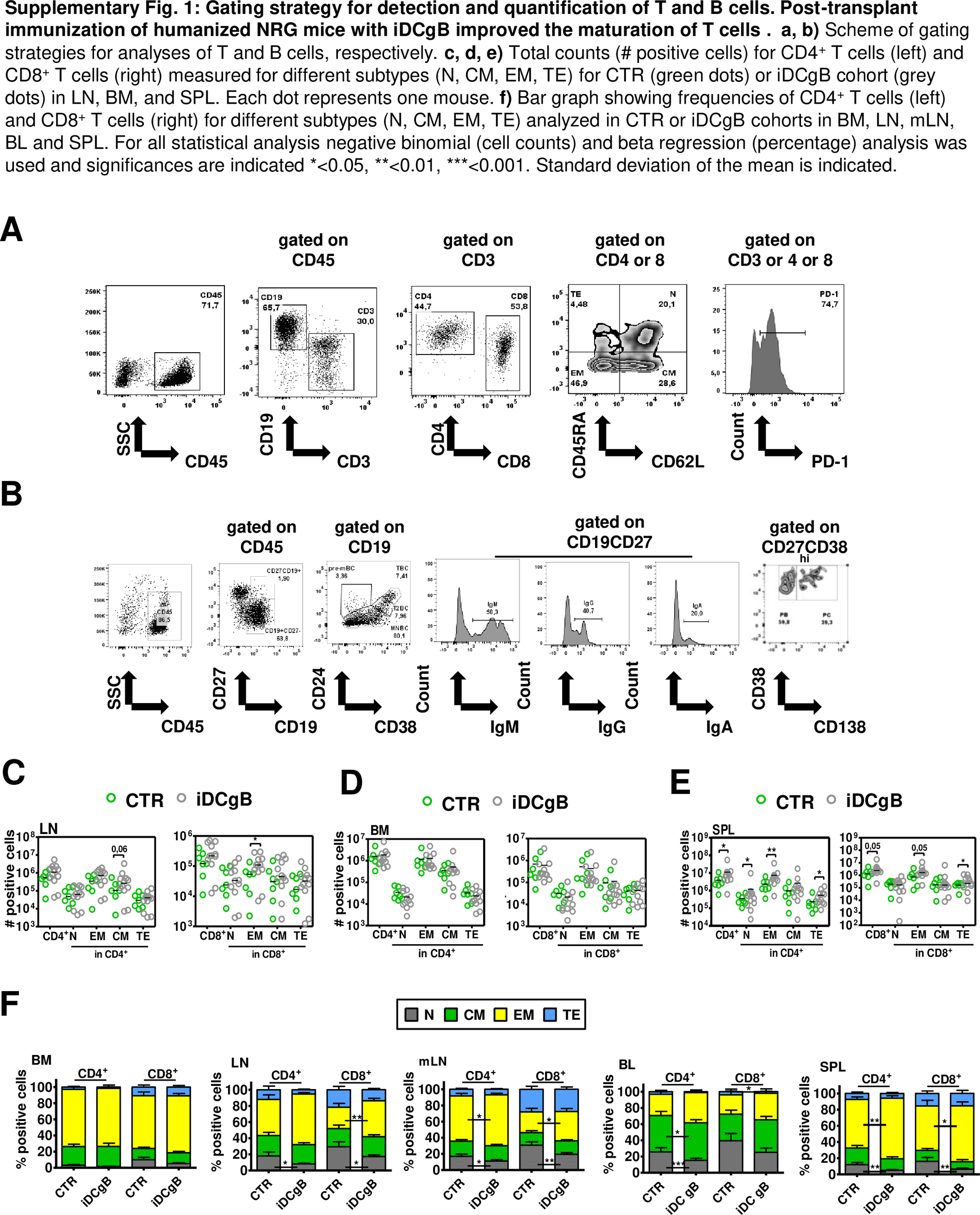

Supplement: S1 Fig — Post-transplant immunization of humanized NRG mice with iDCgB improved the maturation of T cells. (TIF) [file ppat.1008560.s001.tif]

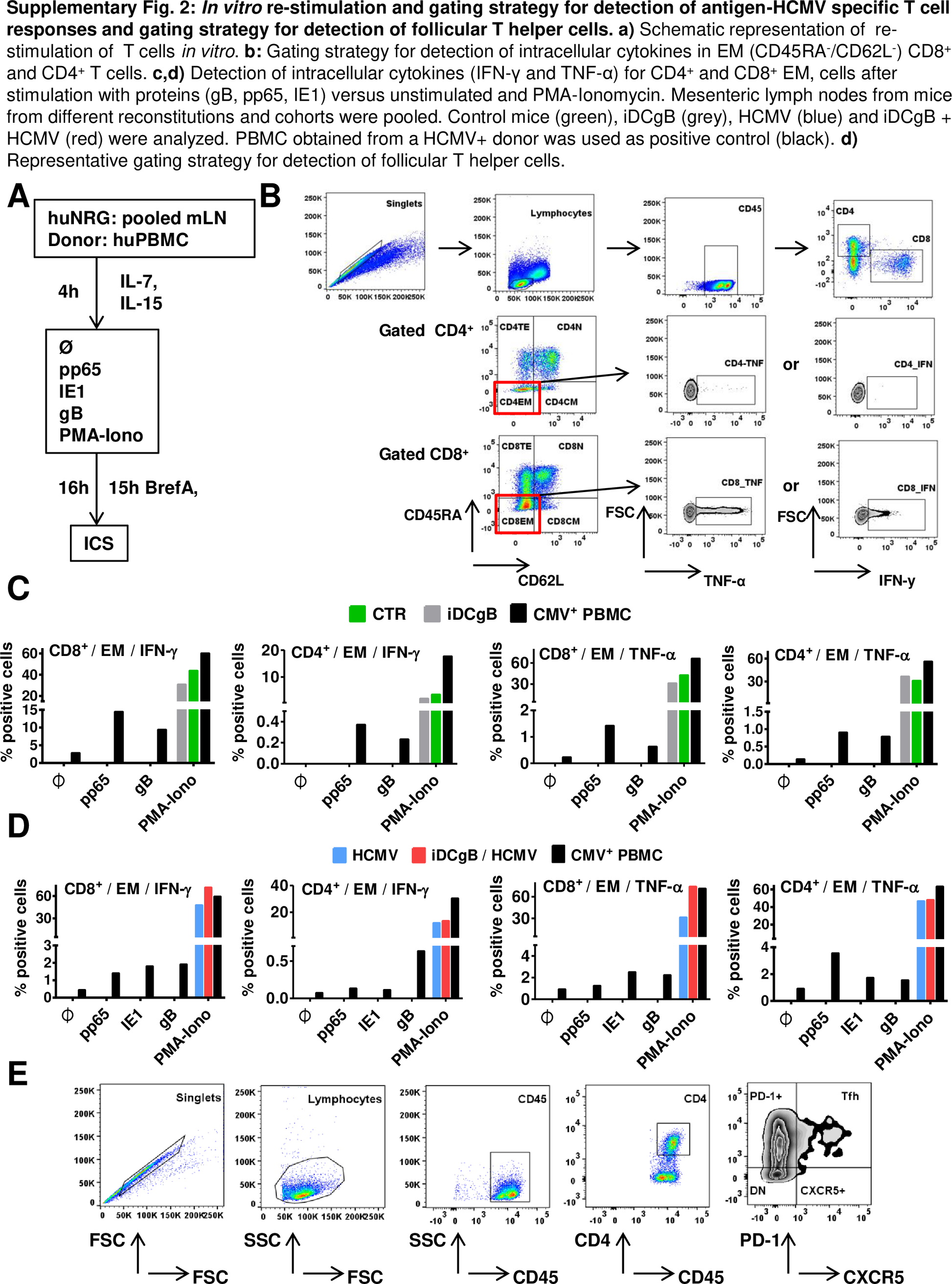

Supplement: S2 Fig — (TIF) [file ppat.1008560.s002.tif]

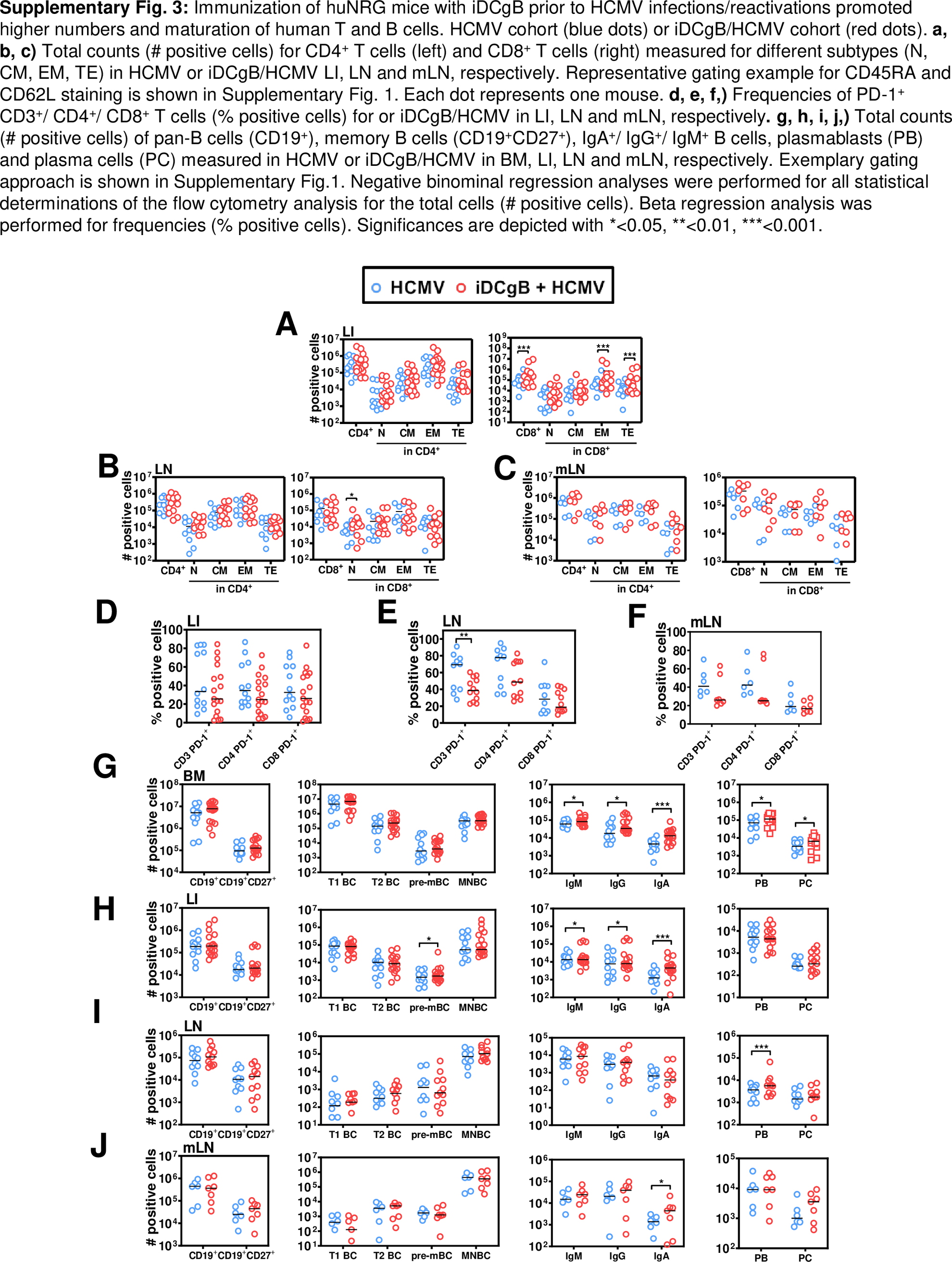

Supplement: S3 Fig — (TIF) [file ppat.1008560.s003.tif]

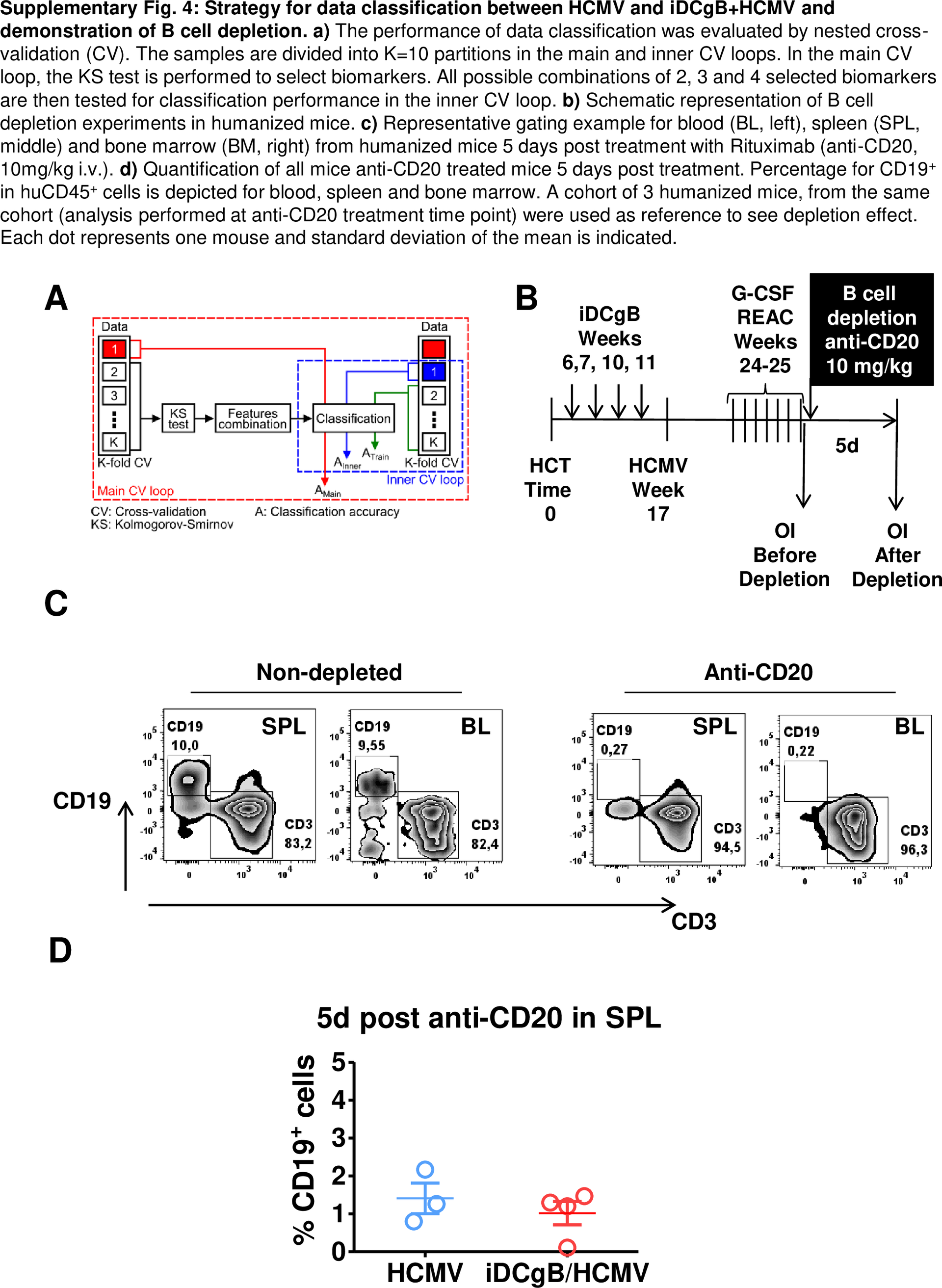

Supplement: S4 Fig — (TIF) [file ppat.1008560.s004.tif]

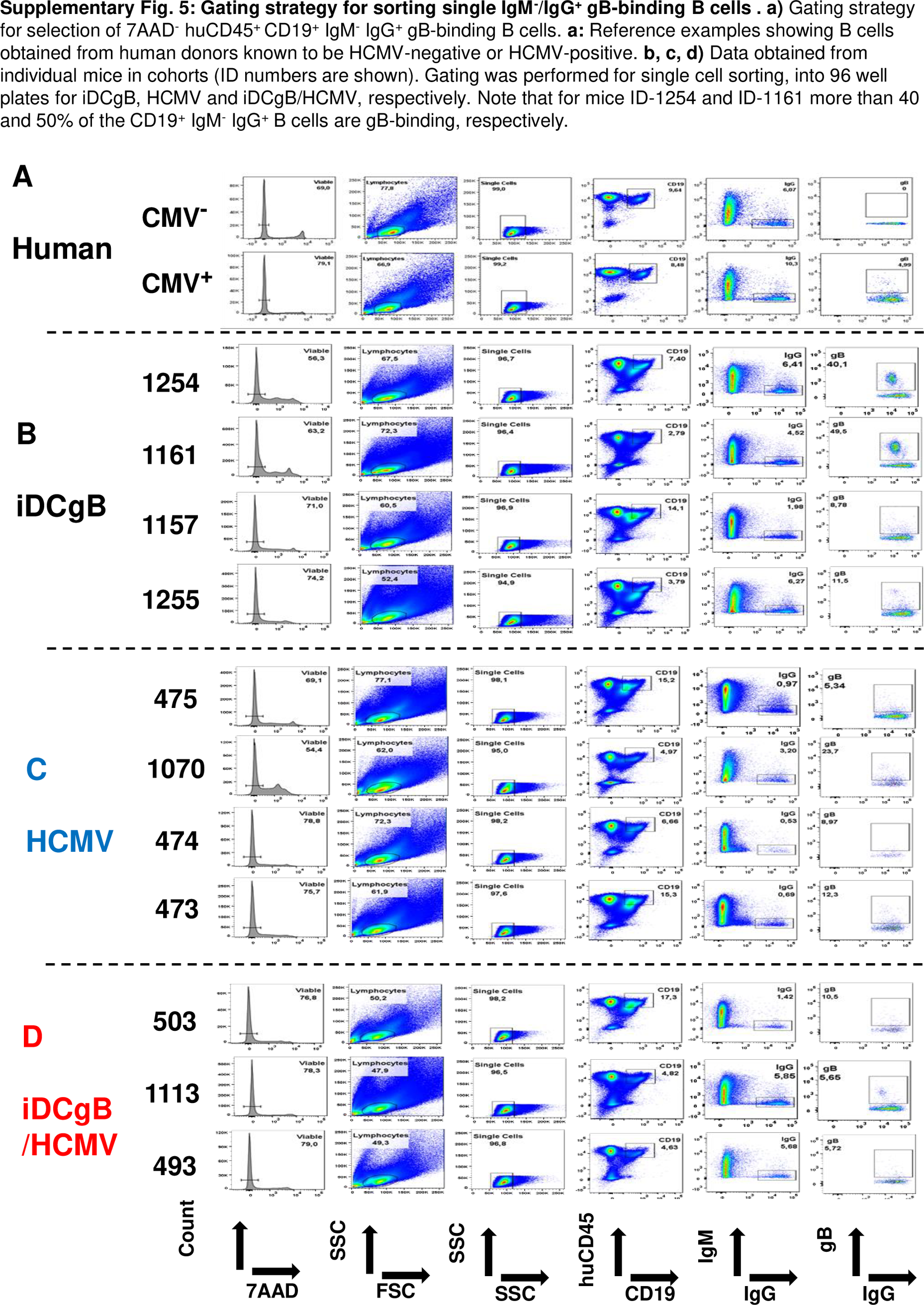

Supplement: S5 Fig — (TIF) [file ppat.1008560.s005.tif]

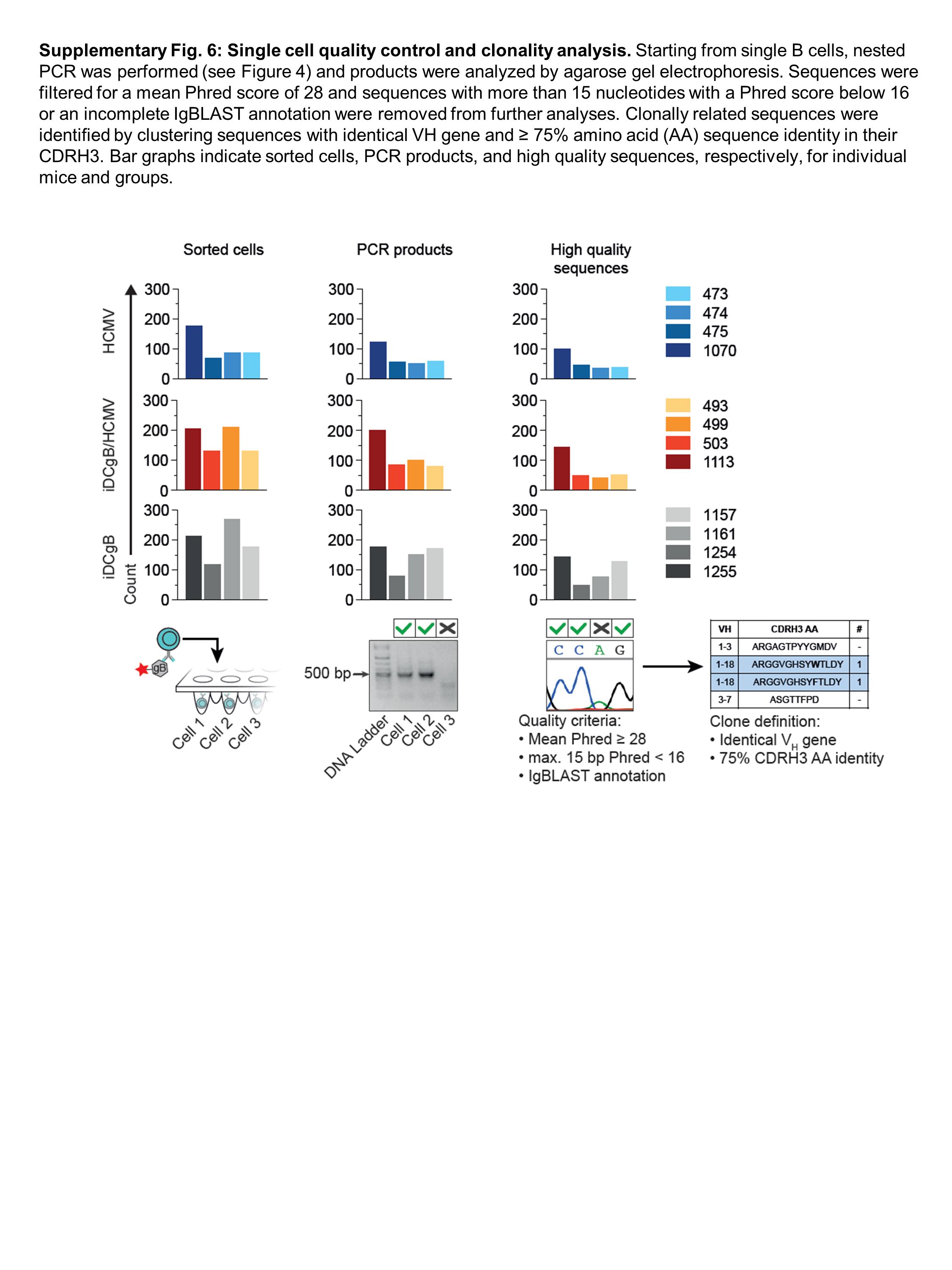

Supplement: S6 Fig — (TIF) [file ppat.1008560.s006.TIF]
