# Supplementary material for: Repertoire characterization and validation of gB-specific human IgGs directly cloned from humanized mice vaccinated with dendritic cells and protected against HCMV
Source: PLoS Pathog. 2020 Jul 15;16(7):e1008560. doi: 10.1371/journal.ppat.1008560 (PMC7363084; doi:10.1371/journal.ppat.1008560)
Supplement: S1 Table — (DOCX) [file ppat.1008560.s007.docx]

**Supplementary Table 1 for data presented in Fig. 1F,J:** Descriptive statistics regarding immune phenotype for SPL and mLN in total numbers and SPL in percentage, LSM: least squares means estimation; RR: rate ratio; OR: odds ratio (between CTR and iDCgB).
