# Supplementary material for: Repertoire characterization and validation of gB-specific human IgGs directly cloned from humanized mice vaccinated with dendritic cells and protected against HCMV
Source: PLoS Pathog. 2020 Jul 15;16(7):e1008560. doi: 10.1371/journal.ppat.1008560 (PMC7363084; doi:10.1371/journal.ppat.1008560)
Supplement: S2 Table — (DOCX) [file ppat.1008560.s008.docx]

**Supplementary Table 2:** Descriptive representation of data shown in Fig.2 regarding the HCMV PCR analyses. For mice immunized with iDCgB and challenged with HCMV, mice were classified as Fully Protected (F-PR, 56,25%) when HCMV genomic copies were not detectable (ND) in liver and as partially protected (P-PR, 37,25%) when HCMV genomic copies detectable in liver were 2 log lower than the average measures for the non-immunized HCMV-challenged mice (8 x10^7^ copies /μg DNA). DNA samples not available for PCR analyses of bone marrow are depicted (N.A.). The frequencies of memory B cells (% CD19^+^ CD27^+^) detectable in spleen are shown for all mice.

| Mouse ID  /  Protection | CB  donor | Cohort | PCR LI (copies/µg DNA) | PCR BM  (copies/µg DNA) | % CD19^+^ CD27^+^ IgG^+^ in SPL |
| --- | --- | --- | --- | --- | --- |
| 1242 | D1 | HCMV | 4,14x10^4^ | N,A, | 1,6x10^4^ |
| 1243 | D1 | HCMV | 1,16x10^5^ | N,A, | 2,1x10^4^ |
| 1246 | D1 | HCMV | 2,37x10^7^ | N,A, | 1,2x10^4^ |
| 1247 | D1 | HCMV | 2,49x10^4^ | N,A, | 2,9x10^4^ |
| 1069 | D2 | HCMV | 1,74x10^4^ | 1,84x10^4^ | 4,3x10^3^ |
| 1070 | D2 | HCMV | 1,90x10^4^ | 9,59x10^3^ | 3,9x10^4^ |
| 1106 | D2 | HCMV | 2,41x10^4^ | 4,02x10^4^ | 1,2x10^3^ |
| 473 | D3 | HCMV | 8,67x10^6^ | 3,04x10^6^ | 2,9x10^5^ |
| 474 | D3 | HCMV | 3,13x10^8^ | 1,14x10^5^ | 1,3x10^5^ |
| 475 | D3 | HCMV | 1,74x10^6^ | 6,45x10^6^ | 1,2x10^5^ |
| 476 | D3 | HCMV | 6,38x10^8^ | 3,20x10^6^ | 1,0x10^5^ |
| 477 | D3 | HCMV | 1,11x10^6^ | 3,38x10^5^ | 1,8x10^5^ |
|  | | ***Mean*** | ***8,22x10^7^*** | ***1,65x10^6^*** | ***7,8x10^4^*** |
|  |  | ***SD*** | ***1,88x10^8^*** | ***2,22x10^6^*** | ***8,4x10^4^*** |
| 1261 /P-PR | D1 | DC+HCMV | 1,45x10^4^ | N,A, | 1,0x10^5^ |
| 1262 /F-PR | D1 | DC+HCMV | N,D, | N,A, | 9,1x10^4^ |
| 1265 /P-PR | D1 | DC+HCMV | 1,41x10^5^ | N,A, | 1,4x10^5^ |
| 1266 /F-PR | D1 | DC+HCMV | N,D, | N,A, | 1,2x10^5^ |
| 1267 /F-PR | D1 | DC+HCMV | N,D, | N,A, | 1,1x10^5^ |
| 1273 /F-PR | D1 | DC+HCMV | N,D, | N,A, | 1,1x10^5^ |
| 1098 /P-PR | D2 | DC+HCMV | 6,16x10^3^ | 1,03x10^3^ | 2,2x10^5^ |
| 1099 /F-PR | D2 | DC+HCMV | N,D, | N,D | 8,7x10^4^ |
| 1100 /P-PR | D2 | DC+HCMV | 3,81x10^3^ | 1,15x10^3^ | 4,2x10^4^ |
| 1113 /F-PR | D2 | DC+HCMV | N,D, | N,D, | 7,5x10^4^ |
| 492 /F-PR | D3 | DC+HCMV | N,D, | N,D | 2,7x10^5^ |
| 493 /F-PR | D3 | DC+HCMV | N,D, | N,D, | 1,9x10^5^ |
| 494 /P-PR | D3 | DC+HCMV | 8,52x10^5^ | 3,05x10^2^ | 1,4x10^5^ |
| 498 | D3 | DC+HCMV | 3,42x10^8^ | 4,23x10^6^ | 3,0x10^5^ |
| 499 /F-PR | D3 | DC+HCMV | N,D, | N,D, | 1,2x10^5^ |
| 503 /P-PR | D3 | DC+HCMV | 1,23x10^3^ | N,D, | 1,7x10^5^ |
| Total: 16 mice analyzed  Full PR:  9 mice - 56,25%  Partially PR: 6 mice - 37,25% | | ***Mean*** | ***2,14x10^7^*** | ***4,23x10^5^*** | ***1,3x10^5^*** |
|  |  | ***SD*** | ***8,28x10^7^*** | ***1,27x10^6^*** | ***6,8x10^4^*** |
