# Supplementary material for: Repertoire characterization and validation of gB-specific human IgGs directly cloned from humanized mice vaccinated with dendritic cells and protected against HCMV
Source: PLoS Pathog. 2020 Jul 15;16(7):e1008560. doi: 10.1371/journal.ppat.1008560 (PMC7363084; doi:10.1371/journal.ppat.1008560)
Supplement: S3 Table — (DOCX) [file ppat.1008560.s009.docx]

**Supplementary Table 3:** Qualitative analyses for detection of human IgG-reactivity in plasma of mice by strips immunoassays. Detection of HCMV antigens were classified as + for clearly positive bands; - for clearly non-detectable bands; +/- = faint bands. Analyses were performed with plasma obtained from mice transplanted with cord blood from different donors and challenged with HCMV (HCMV, blue) or immunized with iDCgB and challenged with HCMV (DC+HCMV, pink and red).

| Mouse ID | Cohort | Group | IE1 | CM2 | p150 | pp65 | gB1 | gB2 |
| --- | --- | --- | --- | --- | --- | --- | --- | --- |
| 1242 | D1 | HCMV | - | - | +/- | +/- | **+/-** | **+/-** |
| 473 | D3 | HCMV | +/- | +/- | + | +/- | **+** | **-** |
| 474 | D3 | HCMV | - | - | - | - | **-** | **-** |
| 475 | D3 | HCMV | - | - | +/- | +/- | **+** | **+** |
| 476 | D3 | HCMV | - | - | - | - | **-** | **-** |
| 477 | D3 | HCMV | - | - | - | - | **-** | **-** |
| 1248 | D1 | DC+HCMV | - | - | - | +/- | **-** | **+/-** |
| 1254 | D1 | DC+HCMV | + | + | + | + | **+** | **+** |
| 1257 | D1 | DC+HCMV | + | + | + | + | **+** | **+** |
| 1262 | D1 | DC+HCMV | - | - | - | - | **-** | **-** |
| 1267 | D1 | DC+HCMV | + | +/- | + | +/- | **+/-** | **+** |
| 492 | D3 | DC+HCMV | - | - | - | - | **-** | **-** |
| 493 | D3 | DC+HCMV | - | - | - | - | **-** | **-** |
| 494 | D3 | DC+HCMV | - | - | - | - | **-** | **-** |
| 498 | D3 | DC+HCMV | - | - | - | - | **-** | **-** |
| 499 | D3 | DC+HCMV | + | + | + | + | **+** | **+** |
| 503 | D3 | DC+HCMV | + | + | + | + | **+** | **+** |
