# Supplementary material for: Repertoire characterization and validation of gB-specific human IgGs directly cloned from humanized mice vaccinated with dendritic cells and protected against HCMV
Source: PLoS Pathog. 2020 Jul 15;16(7):e1008560. doi: 10.1371/journal.ppat.1008560 (PMC7363084; doi:10.1371/journal.ppat.1008560)
Supplement: S7 Table — (DOCX) [file ppat.1008560.s013.docx]

**Supplementary Table 7:** Antibodies used in the studies.

| Antigen | Fluorochrome | Company | Dilution | Catalogue # |
| --- | --- | --- | --- | --- |
| Anti-human IgG | HRP | Roth | 1:1000 | 47531 |
| Anti-mouse IgG | AL647 | Biolegend | 1:250 | 405322 |
| Anti-mouse IgG | AL488 | Biolegend | 1:250 | 405319 |
| CD138 | PE | Miltenyi | 1:100 | 130-101-168 |
| CD14 | FITC | Miltenyi | 1:25 | 130-080-701 |
| CD185 (CXCR5) | AL700 | Biolegend | 1:25 | 356904 |
| CD19 | AL700 | Biolegend | 1:20 | 302226 |
| CD19 | PerCP-Cy5,5 | Biolegend | 1:100 | 302230 |
| CD24 | FITC | BD | 1:100 | 555427 |
| CD27 | APC-Cy7 | Biolegend | 1:100 | 302816 |
| CD3 | BV510 | Biolegend | 1:100 | 300448 |
| CD38 | APC | Biolegend | 1:50 | 303510 |
| CD4 | PerCP | Biolegend | 1:250 | 317432 |
| CD45 | Pacific Blue | Biolegend | 1:100 | 304022 |
| CD45 | AL700 | Biolegend | 1:100 | 304024 |
| CD45RA | FITC | Beckman Coulter | 1:100 | A07786 |
| CD45RA | BV605 | Biolegend | 1:100 | 304134 |
| CD62L | PE-Cy5 | Beckman Coulter | 1:100 | IM26554 |
| CD62L | FITC | BD | 1:25 | 555543 |
| CD69 | APC-Cy7 | Biolegend | 1:100 | 310914 |
| CD8 | PE-Cy7 | Biolegend | 1:100 | 344712 |
| CD8a | PE-Cy7 | Biolegend | 1:100 | 300914 |
| CD279 (PD-1) | PE | Biolegend | 1:100 | 329906 |
| IFN-γ | PE | Biolegend | 1:100 | 502509 |
| IgA | FITC | Thermo | 1:100 | H14001 |
| IgA | PE | Miltenyi | 1:100 | 130-093-128 |
| IgG | PE-Cy7 | BD | 1:50 | 561298 |
| IgG | PE | BD | 1:4 | 555787 |
| IgM | Pacific Blue | Biolegend | 1:100 | 314514 |
| IgM | FITC | BD | 1:3 | 555782 |
| p27-287 (gB) | - | M. Mach | 1:20 | - |
| SM5-1 (gB) | - | M,Mach | 1:1000 | - |
| TNF-α | APC | Biolegend | 1:100 | 502912 |
