# Supplementary material for: Repertoire characterization and validation of gB-specific human IgGs directly cloned from humanized mice vaccinated with dendritic cells and protected against HCMV
Source: PLoS Pathog. 2020 Jul 15;16(7):e1008560. doi: 10.1371/journal.ppat.1008560 (PMC7363084; doi:10.1371/journal.ppat.1008560)
Supplement: S8 Table — (DOCX) [file ppat.1008560.s014.docx]

**Supplementary Table 8:** Descriptive data classification regarding data presented in Fig. 3.

| Biomarkers combination set | Occurrences in 10-fold CV | Avg, A_Train_ | Avg, A_Inner_ | Avg, A_Main_ |
| --- | --- | --- | --- | --- |
| **%-LI IgA, %-SPL IgA, #-SPL IgA** | **9** | **94.52%** | **72.41%** | **74.44%** |
| **#-BM CD4, %-LI IgA, #-SPL IgG** | **8** | **100%** | **74.17%** | **86.67%** |
| **#-BM CD4, %-SPL IgA, #-SPL IgG** | **8** | **93.28%** | **72.29%** | **72.5%** |
| **#-BM CD4, #-BM IgA, %-LI IgA, #-LI IgA** | **8** | **99.52%** | **77.29%** | **78.54%** |
