# Supplementary material for: Repertoire characterization and validation of gB-specific human IgGs directly cloned from humanized mice vaccinated with dendritic cells and protected against HCMV
Source: PLoS Pathog. 2020 Jul 15;16(7):e1008560. doi: 10.1371/journal.ppat.1008560 (PMC7363084; doi:10.1371/journal.ppat.1008560)
Supplement: S9 Table — (DOCX) [file ppat.1008560.s015.docx]

**Supplementary Table 9:** Statistical analyses of B cell depletion with monoclonal antibodies regarding data presented in **Fig. 3**.

**A.** Bioluminescence analyses (Total flux p/s) of HCMV-infected CTR mice before and 5 days after B cell depletion with anti-CD20 mAbs. (NA: Not Applicable).

| **Mice/ Time of analyses** | **CTR baseline** | **CTR**  **after** |
| --- | --- | --- |
| Mouse 1 | 1,036,000 | 1,706,000 |
| Mouse 2 | 1,453,000 | 1,397,000 |
| Mouse 3 | 1,344,000 | 1,168,000 |
| **Mean** | 1,277,667 | 1,423,667 |
| **Median** | 1,344,000 | 1,397,000 |
| **SD** | 216,269 | 216,269 |
| **P value t test** | (NA) | 0.63 |
| **P value Mann-Whitney-Wilcoxon test** | (NA) | 1 |
| **Signal relative to baseline %** | 100% | 104% |

**B**. Bioluminescence analyses (Total flux p/s) of iDCgB immunized and then HCMV-infected mice before and 5 days after B cell depletion with anti-CD20 mAbs. (NA: Not Applicable).

| **Mice/ Time of analyses** | **iDCgB baseline** | **iDCgB**  **after** |
| --- | --- | --- |
| Mouse 1 | 899,700 | 1,083,000 |
| Mouse 2 | 841,900 | 1,083,000 |
| Mouse 3 | 917,200 | 978,100 |
| Mouse 4 | 923,300 | 994,300 |
| **Mean** | 895,525 | 1,034,600 |
| **Median** | 908,450 | 1,038,650 |
| **SD** | 37,122 | 56,277 |
| **P value t test** | (NA) | ***0.051*** |
| **P value Wilcoxon test** | (NA) | 0.125 |
| **Signal relative to baseline %** | 100% | *114%* |
