# Supplementary material for: Repertoire characterization and validation of gB-specific human IgGs directly cloned from humanized mice vaccinated with dendritic cells and protected against HCMV
Source: PLoS Pathog. 2020 Jul 15;16(7):e1008560. doi: 10.1371/journal.ppat.1008560 (PMC7363084; doi:10.1371/journal.ppat.1008560)
Supplement: S10 Table — (DOCX) [file ppat.1008560.s016.docx]

**Supplementary Table 10:** Statistical analyses of passive immunization with monoclonal antibodies regarding data presented in **Fig. 5**.

**A.** Bioluminescence analyses (Total flux p/s) of control HCMV-infected mice vs. mice pre-treated with mAbs and then HCMV-infected. (NA: Not Applicable).

|  | **Mice/ Cohorts** | **CTR**  **HCMV** | **mAbs**  **HCMV** |
| --- | --- | --- | --- |
|  | Mouse 1 | 3,090 | 1,634 |
|  | Mouse 2 | 2,599 | 1,634 |
|  | Mouse 3 | 2,365 | 1,997 |
|  | **Mean** | 2,684 | 1,755 |
|  | **Median** | 2,599 | 1,634 |
|  | **SD** | 370.013 | 209.578 |
|  | **P value t test** | (NA) | ***0.0294*** |
|  | **P value Wilcoxon test** | (NA) | 0.0765 |
|  | **Signal relative to CTR** | 100% | ***62.8%*** |

**B.** Bioluminescence analyses (Total flux p/s) of control HCMV-infected/ reactivated mice vs. mice pre-treated with mAbs and then HCMV-infected/ reactivated. (NA: Not Applicable).

|  | **Mice/ Cohorts** | **CTR**  **HCMV/**  **REAC** | **mAbs**  **HCMV/**  **REAC** |
| --- | --- | --- | --- |
|  | Mouse 1 | 2,943 | 1,957 |
|  | Mouse 2 | 2,970 | 1,916 |
|  | Mouse 3 | 3,387 | 3,513 |
|  | **Mean** | 3,100 | 2,462 |
|  | **Median** | 2,970 | 1,957 |
|  | **SD** | 248.915 | 910.423 |
|  | **P value t test** | (NA) | 0.348 |
|  | **P value Wilcoxon test** | (NA) | 0.0700 |
|  | **Signal relative to CTR** | 100% | ***65.9%*** |

**C.** RT-q-PCR analyses (copies/µgDNA) of liver of control HCMV-infected/ reactivated mice vs. mice pre-treated with mAbs and then HCMV-infected/ reactivated. (NA: Not Applicable).

|  | **Mice/ Cohorts** | **CTR**  **HCMV**  **REAC** | **mAbs**  **HCMV**  **REAC** |
| --- | --- | --- | --- |
|  | Mouse 1 | 463,503.60 | 20,143.32 |
|  | Mouse 2 | 53,808.75 | 63,248.49 |
|  | Mouse 3 | 1,721,405.00 | 87,820.84 |
|  | **Mean** | 746,239.10 | 57,070.88 |
|  | **Median** | 463,503.60 | 63,248.49 |
|  | **SD** | 869,007.40 | 34,259.07 |
|  | **P value t test** | (NA) | 0.303 |
|  | **P value Wilcoxon test** | (NA) | 0.400 |
|  | **Signal relative to CTR** | 100% | ***14%*** |

**D**. RT-q-PCR analyses (copies/µgDNA) of bone marrow of control HCMV-infected/ reactivated mice vs. mice pre-treated with mAbs and then HCMV-infected/ reactivated. (NA: Not Applicable).

|  | **Mice/ Cohorts** | **CTR**  **HCMV**  **REAC** | **mAbs**  **HCMV**  **REAC** |
| --- | --- | --- | --- |
|  | Mouse 1 | 374,934.80 | 4,252,498.00 |
|  | Mouse 2 | 42,715.87 | 59,940.32 |
|  | Mouse 3 | 280,346.00 | 89,083.70 |
|  | **Mean** | 232,665.60 | 1,467,174.00 |
|  | **Median** | 280,346.00 | 89,083.70 |
|  | **SD** | 171,164.90 | 2,412,205.00 |
|  | **P value t test** | (NA) | 0.469 |
|  | **P value Wilcoxon test** | (NA) | 1 |
|  | **Signal relative to CTR** | 100% | ***32%*** |
