# Supplementary material for: Repertoire characterization and validation of gB-specific human IgGs directly cloned from humanized mice vaccinated with dendritic cells and protected against HCMV
Source: PLoS Pathog. 2020 Jul 15;16(7):e1008560. doi: 10.1371/journal.ppat.1008560 (PMC7363084; doi:10.1371/journal.ppat.1008560)
Supplement: Attachment — Submitted filename: Theobald Rebuttal letter .pdf [file ppat.1008560.s017.pdf]

MHH

Medizinische Hochschule Hannover

Zentrum Innere Medizin
Hämatologie, Hämostaseologie und Onkologie
Und Stammzelltransplantation
Prof. Dr. med. Arnold Ganser, Direktor

MHH - Hämatologie, Hämostaseologie und Onkologie - 30623 Hannover

Regenerative Immune Therapies Applied
Prof. Dr. Renata Stripecke
Telefon: ++49 (0) 511 532-6999
Fax: ++49 (0) 511 532-6975
Stripecke.renata@mh-hannover.de
www.stripecke-mhh.de

Carl-Neuberg-Straße 1 OE6860
30625 Hannover
Telefon: 0511 532-0
www.mh-hannover.de

Grant McFadden

Kasturi Haldar

Editors-in-Chief

PlosPathogens

Ref: PPATHOGENS-D-19-01985

28. Februar 2020

Dear Dr. McFadden and Dr. Haldar, dear Guest and Section Editors, dear Reviewers,

Thank you very much for your interest and insightful questions to improve the quality of our work.

Here, we provide a major revision of our paper retitled to: ***"Repertoire characterization and validation of gB-specific human IgGs directly cloned from humanized mice vaccinated with dendritic cells and protected against HCMV"***. As the title was not clear for one reviewer, we can propose another alternative title: *"Dendritic cells expressing gB elicit potent and long-lasting humoral protection against HCMV reactivation in humanized mice and enable the generation of gB-specific monoclonal antibodies".*

We obtained extensive additional corroborating data: (i) We addressed the main request of the editors and reviewers regarding the validation of the cloned monoclonal antibodies through passive immunization against HCMV and the results are conclusive; (ii) We performed B cell depletions to emphasize the relevance of B cells in the HCMV control; (iii) As inquired by all reviewers, we analyzed antigen-specific T cell responses and we obtained definite *in vitro* functional data. We optimized the conversion of the figures and we hope that they are satisfactory. The main revisions in the manuscript are highlighted and we hope that this version and point-by-point responses will clarify the concerns from the editors and reviewers.

Since the reviewers inquired about several aspects of the humanized mouse models, we are attaching for their consultation:

1. Our paper describing the HCMV/GLuc reactivation in a dynamic humanized mouse model system (Theobald et al, 2018, Front. Immunol),
2. Our recent review regarding humanized mouse models for testing HCMV vaccines (Koenig et al, 2020, Vaccines).

We addressed the reviews' major and minor concerns, included additional references and amended the corrected legends. These revisions are highlighted in the marked version. Additionally, we re-revised and edited the manuscript thoroughly for minor grammar and spelling mistakes, and these smaller corrections are not marked. Below is a point-by-point response to the Reviewers. All the co-authors have approved the submission of the revised paper.

If the initial reviewers are not available for this second review, I would kindly ask you to exclude investigators from the Vaccine and Gene Therapy Institute, Oregon Health & Science University (Drs. Nelson, Caposio and colleagues) due to potential conflicts of interest.

We hope that this major revision addressed the critical points raised by the reviewers. If you have any further questions or requests, please do not hesitate to contact me at any time.

Sincerely yours,

Renata Stripecke

Prof. Dr. Renata Stripecke
Department of Hematology, Hemostasis,
Oncology, and Stem Cell Transplantation
Hannover Medical School, Germany

<u>Part I – Summary</u>

<u>Reviewer #1:</u> While the presented results are in line with several recent studies that suggest IgG antibody production after immunization in humanized mice, their role in the observed immune control of HCMV should be better characterized. The study suggests that protective antibody responses can be induced. However, this suggestion should be proven either by B cell depletion or adoptive antibody transfer.

We thank this reviewer for raising these important points. We addressed these requirements with additional gain or loss of function *in vivo* experiments:

Adoptive antibody transfer. This major concern was also recommended by reviewers 2 and 3. For passive immunizations, we used the two recombinant monoclonal antibodies showing the highest binding to surface gB (DC06 and PR32). Administration of the antibodies prior to HCMV challenge and then continuously until the reactivation period resulted into lower levels of HCMV detection by optical imaging analyses and PCR. See data: in **Fig 5 n-p,** description of results (lines 351-371), discussion (lines 455-463) and methods (lines 732-740).

B cell depletion. This experiment was suggested by this reviewer only. As B cell depletion is not a routine approach performed in humanized mice, we searched the literature and obtained kind guidance for the experimental design from Dr. Kristina Howard (FDA). Interestingly, CD20 depletion interfered with the effects of the iDCgB immunization and was associated with higher levels of HCMV detection. See data in **Fig 3 e-f**, description of results (lines 284-295), discussion (lines 396-399) and methods (lines 732-740).

<u>Reviewer #2:</u> The studies in general look to be have been well done. There are a lot of data presented and sometimes it is hard to pick out the key data as the presentation could have been better (it may be my pdf viewer but some of the graphs were blurred making it hard to visualise some of the data points).

We apologize for the previous data presentation. The figures display the key data-sets chosen for a thematically coherent topic. Additional experimental information or complementary data are shown as supplementary figures or in supplemental tables. We revised the text thoroughly to facilitate the flow and understanding of each experimental milestone. The “Results” section was edited and each sub-topic is now addressed in separate subheadings. We also tried to optimize the conversion of the figures to the PlosPathogens uploading system and we hope they are improved now.

One aspect was that it was somewhat surprising that no discussion in light of the multiple attempts to use gB as a vaccine in humans and other animal models (and

***downstream attempts to understand how those vaccines worked) have been published but little mention is made of them. I am thinking of work from Adler, Bernstein, Schleiss, Diamond, McAvoy, Griffiths, Reeves, Permar etc that all have investigated the basis of immune responses against gB. Parallels could easily be drawn*.**

We apologize for this oversight. We added this information to the discussion (lines 375-382).

Part II – Major Issues: Key Experiments Required for Acceptance

Reviewer #1:

In order to demonstrate protection by the elicited humoral immune response, gain- or loss-of-function experiments should be performed. The authors could either deplete B cells with anti-CD20 antibodies to possibly abrogate protection against HCMV infection or adoptively transfer one of their isolated neutralizing antibodies into HCMV infected humanized mice to demonstrate that these antibodies mediate immune control in vivo.

These were very good suggestions and the key experiments were performed as described above (**Fig 3 e-f**).

The authors demonstrate that their iDC-gB treatment increases CD4+ T cell numbers and especially in the bone marrow these correlate with protection from HCMV challenge, but they never attempt to demonstrate HCMV or even gB specificity of these T cell populations.

a. Do these CD4+ T cells produce cytokines in response to re-stimulation with HCMV antigens?

We thank the reviewer for this question, which was also asked by the other two reviewers. Yes, we observed $CD4^+$ T cell responses against HCMV antigens. T cell re-stimulation *in vitro* with recombinant proteins (gB, pp65 and IE1) and intracellular analyses for detection of IFN-γ and TNF-α expression were performed. This was done in collaboration with Dr. Agnes Bonifacius and Prof. Britta Eiz-Vesper. The data are presented as:

- T cell responses after iDCgB vaccination: **Fig. 1h**, description of results (lines 160-175), discussion (lines 391) and methods (lines 600-623).
- T cell responses after iDCgB vaccination and HCMV reactivation: **Fig 2i** , description of results (lines 227-239), discussion (lines 391) and methods (lines 600-623).

b. Is especially IL-21 production observed?

This is an interesting question. Unfortunately, we were not able to perform analyses of IL-21 since there were not sufficient remaining T cells recovered from the lymph nodes of humanized mice. Further, this test is not standard in most laboratories and would have to be properly set up.

c. Are follicular helper CD4+ T cells increased in number after iDCgB treatment?

Thanks for this question. Yes, we were able to analyze and quantify follicular helper $CD4^+$ T cells obtained from lymph nodes from control and iDCgB immunized mice. The data is presented in **Fig. 1g** and the description of these results in lines157-160.

3. In order to judge the potency of the described method to induce IgG responses some additional characteristics of the cloned antibodies should be revealed. Even so it is mentioned in the abstract that only low to moderate levels of somatic hypermutation were observed, no concrete numbers seem to be given in the manuscript.

We thank the reviewer for this important question. We would like to point to **Fig 4** in terms of the overall antibody response. As a measure of somatic hypermutation, we provided the information of the VH gene germline identity (**Fig 4f**). Here, we reported a mean germline identity of about 95 % (line 313 of the revised manuscript). As the reference (dashed line) we also provided the mean germline identity of 6 healthy individuals (characterized from subjects reported in Ehrhardt et al., 2019), showing an almost identical distribution. We thus concluded that there were only low to moderate levels of SHM in the overall repertoire.

Especially the number of somatic hypermutations in the isolated nine antibodies should be reported.

The germline identity information of the cloned and tested antibodies was provided in **Table 1**. However, since germline identity refers to nucleotides and thus includes both, synonymous and non-synonymous mutations, we revised **Table 1** and now included the count of somatic hypermutations on an amino acid level to account only for non-synonymous mutations.

Do these correlate with gB binding affinity or neutralization capacity?

We did not find any correlation between sequence features and affinity or neutralizing capacity. We included a section to improve the clarity regarding the sequence features listed in **Table 1** (lines 343-348 of the revised manuscript)**.**

Reviewer #2:

1. One major point that needs clarification is the measure of control. My understanding is that the mice are injected with humanised CD34+ cells, vaccinated with the iDCgB, and then challenged with MRC5 cells infected with HCMV. I presume the premise is that the HCMV will then go latent in the CD34+ cells and thus the authors measure reactivation (similar to the model used by Nelson and colleagues).

a. My question is the establishment of latency the same in all mice? b. Is this measured?

We thank the reviewer for this question. Yes, we see reproducible HCMV infection and latency and reactivations in all mice. We revised the text (lines 90-107) to explain better the status quo of humanized mouse models of HCMV infection (Koenig et al 2020) and the model described in our lab (Theobald et al 2018). For the current work, we decided to focus on the HCMV reactivation model because reactivation is the main clinical problem and it is associated with immune dysregulations.

c. Hypothetically, the MRC5 cell infection is controlled better in the vaccinated mice? If this led to less latency then it would read out as less reactivation. so the control is of the challenge not reactivation? could the authors clarify?

We understand the reviewer's concern. We performed iDCgB immunization at early time-points after HCT and before the HCMV challenge because we wanted to primarily address if immune protection of an immune compromised host was feasible. Nonetheless, at a later time point in the working model, when the $CD20^+$ B cells were immune-depleted from the mice, we observed a HCMV reactivation rebound. B cell depletion studies were requested by Reviewer 1. Please see data in **Fig 3 e-f**.

d.this is related to point 3 below where it is important to know if the antibodies are controlling initial viraemia or controlling reactivation?

As shown by the additional data obtained for passive immunization, the monoclonal antibodies seemed able to control both the viraemia from the challenge and the reactivation of viraemia occurring due to G-CSF administration. See data: in **Fig 5 n-p**.

e. It is possible that in different settings different immune responses play a role? e.g. reactivation in DCs would be considered to be more cell associated and more resistant to antibodies whereas MRC5s likely make cell free virus where neutralising abs would be more effective? (the authors state work from Stanton's lab so are aware of this aspect of CMV biology)

Sure, we also think that the setting plays a role for immunization, and particularly for immune compromised patients who commonly do not respond well to vaccines. In this work, we proposed to examine specifically the iDCgB immunization approach, with the rationale that the gB trimer formed with its natural conformation on the cell surface would activate the BCR, concurrently with the iDC expression of costimulatory signals and MHC molecules. According to the results obtained with the humanized mice, a potent immune response against gB was enough to provide >90% protection. Nonetheless, we agree that the cell-to-cell spread is a pending issue that remains to be addressed by iDC vaccines incorporating additional antigens (e.g. gH/gL). Please see additional text in the discussion (lines 463-473).

2. Leading on from that is it possible to challenge the mice with MCMV? Presumably the gB immune response will work against MCMV? this would allow the authors to directly analyse primary infection

We thank the reviewer to the interesting basic biology question and suggestion. For our translational work are interested in further refining the HCMV humanized mouse model because we believe that these models will become *per se* relevant for preclinical testing of human vaccines and therapies. We refer the reviewer to our recent review paper regarding *in vivo* models of HCMV infection (Koenig et al, 2020 Vaccines), which is a thriving field and very likely to soon become the state-of-the-art modality .

3. Finally, the authors show evidence of neutralisation. A report in Science suggested the control of CMV reactivation by antibodies was due to non-neutralising functions of antibodies.

We thank the reviewer for this insight. We amended this information in the discussion (lines 455-463).

a. The authors imply the neutralising response is not uniform from all mice so is it possible to correlate neutralising capacity with viraemia/reactivation in the mice?

We apologize to the reviewer, but we did not have enough quantities of mouse plasma to perform these correlative studies.

b. Alternatively can the antibodies be used to provide passive immunity in the model?

Yes, this was addressed for the other reviewer, please see above. As shown by the additional data obtained for passive immunization, the two selected monoclonal antibodies were able to control HCMV. See data: in **Fig 5 n-p**.

Reviewer #3:

1.The authors nicely show the generation of specific antibodies. However, testing of these antibodies is limited to in vitro experiments. In vivo analysis would be important to support the conclusion and relevance stated by the authors.

Yes, this was addressed for the other reviewer, please see above. As shown by the additional data obtained for passive immunization, the two selected monoclonal antibodies were able to control HCMV. See data: in **Fig 5 n-p**.

2. The reader would be interested in experiments combining induction of a T cell response (eg against pp65) together with the iDCgB.

As mentioned before to Reviewer 2, "In this work, we proposed to examine specifically the iDCgB immunization approach, with the rationale that the gB trimer formed with its natural conformation on the cell surface would activate the BCR, concurrently with the iDC expression of costimulatory signals and MHC molecules". An iDCpp65 cell vaccine expressing pp65 was previously developed in our laboratory to stimulate T cell responses against pp65. This iDCpp65 vaccine stimulated both $CD8^+$ and $CD4^+$ T cell reconstitution and responses in humanized mice (Salguero et al, 2014, Daenthasanmak et al, 2015, Volk et al, 2017). As an unanticipated observation of this work, we noticed that iDCpp65 also stimulated lymph node regeneration, remarkable B cell responses and development of IgM and IgG antibodies against pp65. It has been long known that humans develop antibodies against pp65 (see 1991 Ohlin et al Clin. Exp. Immunology https://www.ncbi.nlm.nih.gov/pmc/articles/PMC1535436/) but their immunologic meaning or clinical value to protect against HCMV has not been clarified.

Nonetheless, the reviewer is right that the combination of gB with pp65 as antigens in iDCs could make sense to further improve B and T cell protection and this remains to be done to evaluate additive or synergistic effects. The combination of gB and pp65 has been reported by several groups for example as done for a DNA vaccine reported in 2015 McVoy et al. Vaccine (https://www.ncbi.nlm.nih.gov/pubmed/26597035/), suggesting that gB was sufficient for protection. As mentioned in the abstract of the McVoy paper: *"Importantly, gB-induced epithelial entry neutralizing titers were substantially higher than activities induced by UL130, and both fibroblast and epithelial entry neutralizing titers induced by gB alone as well as gB/pp65 or gB/UL130/pp65 combinations were comparable to those observed in sera from humans with naturally-acquired CMV infections. These findings support further development of Vaxfectin(®)-formulated gB-expressing DNA vaccine for prevention of congenital CMV infections."*

3. What is the translational perspective for the approach? Currently hyperimmunoglobulin agents against HCMV have been shown to have low or absent efficacy. They are not recommended in evidence based recommendations. However,

these clinically available agents are of poor quality and specificity. The authors should provide in vivo evidence, that their identified antibodies are superior to these agents.

We thank the reviewer for this clarification. We described the translational perspective in the discussion (lines 411-426 and 464-472). There are two translational perspectives: (i) the iDCgB immunization for immune compromised hosts on its own to stimulate protective humoral responses against HCMV (which is what we were able demonstrate in our model), and (ii) The development of novel antibodies that can be generated from the humanized mice for passive immunization. We understand the reasoning of the reviewer that comparison with clinically used antibodies will be necessary. However, we have not yet saturated the analyses of monoclonal antibodies that are currently being generated in our humanized mouse model. Since these models are very demanding and costly, such comparison will be made once we have several candidates that can be tested alone or in combinations.

4. The authors correctly stated, that antibodies and T-cell responses are responsible for protective immunity against HCMV. Do iDCgB induce specific T helper cell responses?

Yes, iDCgB induced T helper responses against gB. We performed *in vitro* re-stimulation and intracellular analyses of IFN-γ and TNF-α in $CD4^+$ and $CD8^+$ T cells. The data is presented in **Fig. 1h** and **Fig 2i**.

<u>Part III – Minor Issues:</u>

<u>Reviewer #1:</u>

1. In the title the authors state that they analyzed functional human IgGs. Are there non-functional IgGs? What function do they mean? I would rather specify that they characterized HCMV gB specific human IgGs.

From a total of 9 monoclonal antibodies cloned, produced and analyzed for function, 6 were able to bind to gB. We defined function primarily by gB-binding and, secondarily, if these antibodies neutralized HCMV infection *in vitro*.

We thank the reviewer for this suggestion. We made a small adjustment to: “Repertoire characterization and validation of gB-specific human IgGs directly cloned from humanized mice vaccinated with dendritic cells and protected against HCMV”.

An alternative “Dendritic cells expressing gB elicit potent and long-lasting humoral protection against HCMV reactivation in humanized mice and enable the generation of gB-specific monoclonal antibodies”.

Reviewer #2:

a. A minor issue is whether all the CD34+ cells were from HCMV seronegative donors? Can this be confirmed in the manuscript as of course that could impact on the response observed?

Cord blood donors are typically considered HCMV seronegative donors. The cord blood used for generation of humanized mice was obtained in collaboration with Prof. von Kaisenberg from the department of Obstetrics at our institution. The incidence of congenital HCMV infection in Germany is below 1% and therefore it is unlikely that the cells used in humanized mice were previously infected with HCMV. Not all neonates are tested after birth for congenital HCMV infection, as this is not mandated by the clinical guidelines in Germany. The following text was added to the manuscript (lines 500-502): “Cord blood was obtained from mothers without complications such as acute HCMV infection or reactivation during pregnancy. Neonates were born at term and did not present clinical signs of fetal or congenital HCMV infection”.

b. also were T cell responses against gB detected? I presume they were made. Have the authors ruled out T cell responses as contributing to control?

We thank the reviewer for this relevant question, which was addressed for reviewer 1 above. We performed analyses of T cell responses and long-term effector memory CD8+ and CD4+ T cell responses against gB were observed in the mice. The data is presented in **Fig. 1h** and **Fig 2i**.

Removal from data not shown from the manuscript:

- Line 648: Corrected to: This protein production technique results in the assembly of the trimeric gB observed by size exclusion chromatography.

- Line 744: For one experiment, iDCgB was generated after transduction with a tricistronic vector encoding for GM-CSF/INF-α and gB, which resulted into the same typical co-expression of DC markers and gB (**Fig.1a**).

-Line 1021: Scheme of iDCgB generation. Lentiviral vectors (LV-GMCSF-2A-IFNα + LV-gB or LV-GMCSF-2A-IFNα-2A-gB) were used to transduce CB-CD14+ monocytes and after 16 h the cells were washed and cryopreserved. After thawing, the cells were maintained in culture for seven days. Right panels: Analyses of iDCgB (in this case two LVs were used for co-transduction) by flow cytometry analyses confirming the DC identity (HLA-DR+/CD80+, HLA-DR+/CD86+) and expression of gB on the cell surface. A representative example is shown from more than three experiments for iDCgB generated with two vectors or one tricistronic vector

Line 1204 Corrected to: Wells coated with protein lysates obtained from control 293T/w.t. cells were included in the ELISA assay as negative control.
