# Supplementary material for: Repertoire characterization and validation of gB-specific human IgGs directly cloned from humanized mice vaccinated with dendritic cells and protected against HCMV
Source: PLoS Pathog. 2020 Jul 15;16(7):e1008560. doi: 10.1371/journal.ppat.1008560 (PMC7363084; doi:10.1371/journal.ppat.1008560)
Supplement: Attachment — Submitted filename: Theobald R2 PlosPathogens Rebuttal.doc [file ppat.1008560.s018.doc]

MHH - Hämatologie, Hämostaseologie und Onkologie - 30623 Hannover

**Zentrum Innere Medizin**

**Hämatologie, Hämostaseologie und Onkologie**

**Und Stammzelltransplantation**

**Prof. Dr. med. Arnold Ganser, Direktor**

Regenerative Immune Therapies Applied

Prof. Dr. Renata Stripecke

Telefon:	++49 (0) 511 532-6999

Fax:	++49 (0) 511 532-6975

[Stripecke.renata@mh-hannover.de](mailto:Stripecke.renata@mh-hannover.de)

www.stripecke-mhh.de

Carl-Neuberg-Straße 1 OE6860

30625 Hannover

Telefon: 0511 532-0

www.mh-hannover.de

**Grant McFadden**

**Kasturi Haldar**

**Editors-in-Chief**

**PlosPathogens**

**Ref: PPATHOGENS-D-19-01985-R2**

**30. November 2026**

Dear Dr. McFadden and Dr. Haldar,

dear Guest and Section Editors, dear Reviewers,

Thank you very much for your review, your interest, and for the very reasonable and constructive additional minor critiques and recommendations. Our revisions are provided point-by-point below and they are highlighted in the text: for reviewer 1 in yellow and for reviewer 2 in green.

We hope that Reviewer 1 will find this detailed revision regarding the statistical analyses of the text, figures and supplemental material more comprehensible and acceptable.

In addition, we thank Reviewer 2 for the important insight regarding immune control of latency, which is indeed an important aspect. We would like to mention a recent paper from our group that just came out in “Human Gene Therapy” exploring this humanized mouse model for testing of therapeutic gB-CAR-T cells in the setting of a pre-established HCMV infection. <https://www.liebertpub.com/doi/10.1089/hum.2019.149>.

If you have any additional concerns please do not hesitate to contact me.

Sincerely yours,

**Prof. Dr. Renata Stripecke**

**Department of Hematology, Hemostasis,**

**Oncology, and Stem Cell Transplantation**

**Hannover Medical School, Germany**

**Part I – Summary**

**Editor:**

***1.1. The reviewers have found this study to be significant and important and are supportive of its publication. Reviewer 2 raises an important point for clarification in the text about the level of latency between vaccinated and unvaccinated mice prior to reactivation studies.***

This is a valid concern as immune control of HCMV latency is a very important immune-biological and clinical topic.

The Introduction regarding the humanized mouse model was explained **(lines 105-123)**:

In line with the model described Nelson and Caposio (17), in our humanized mouse model the infection leads primarily to viral latency. In these HCMV-infected mice, reactivation could be induced seven weeks later by daily administrations of Granulocyte-Colony Stimulating Factor (G-CSF). This “reactivation”, analyzed a week after G-CSF treatment was initiated, produced a significant elevation of the optical imaging values analyzed eight weeks later relative to infection without G-CSF treatment (16). Detection of HCMV viral copies by PCR showed that HCMV was detectable after infection in CD34^+^ HSCs and CD14^+^ monocytes, whereas after G-CSF treatment and reactivation the viral copies were conspicuously more abundant in CD34^+^ HSCs and CD169^+^ macrophages (16). Although no specific viral latency marker was evaluated, the infection without final G-CSF treatment showed characteristics of viral latency. Infections were further correlated with development of memory CD4^+^ T cells, whereas reactivation induced an increase of PD-1^+^ activated/ exhausted T cells (16). This model also showed that HCMV reactivation was associated with significant maturation of human IgG^+^ plasma B cells (16). Other noteworthy HCMV infection models have been reported (11), but the advantage of our model is the non-invasive visualization of the HCMV infection and then spread due to reactivation and the consistency of the human antiviral immune responses. Thus, this model is useful for testing the protective effects of human-specific vaccines or immunotherapies and potentially to evaluate the distinct immunological effects induced by HCMV infection/ latency and reactivation.

The Discussion regarding the relevance of HCMV latency was expanded **(lines 437-457)**:

For the current studies, we opted to evaluate the effects of prophylactic vaccination with iDCgB prior to HCMV infection, because we were primarily interested in the early immune reconstitution and antiviral responses shortly after HCT. However, from the clinical perspective, the highest risk group is when the HCT donor is HCMV-seronegative and the recipient is seropositive. In this case, the latent viral reservoir of the recipient can disseminate during the early stages of immune reconstitution, when the recipient is immune compromised, causing serious HCMV disease. Therefore, there is an important need and a large interest to develop novel immunotherapeutic strategies against HCMV latent infections (recently reviewed in ref. (44)). Since during latency the viral gene expression is largely suppressed, it is more challenging to expect immune responses against latency-associated genes (44). Nonetheless, cytotoxic CD4^+^ T cells producing IL-10 and directed against the latency proteins LUNA and UL138 have been detectable in healthy HCMV-positive donors (45). Thus, the T cell responses directed to latency-associated antigens seems to be skewed and rather becomes immunosuppressive, as a mechanism that could sustain viral latency.

The therapeutic effects of iDCgB Immunization after establishment of HCMV latency remains to be evaluated (therapeutic setting). To this end, mice would be infected 15 weeks after HCT and immunized with iDCgB at a later time-point. It would be interesting to evaluate if reactive T cells directed against lytic- or latency-associated antigens could be generated. In fact, using humanized mice infected with HCMV at 17-19 weeks after HCT, we recently showed therapeutic effects of adoptive T cells expressing an engineered chimeric antigen receptor targeting gb (gB-CAR-T) administered 8 weeks later (46).

***1.2. Reviewer 1 raises some concerns about the statistical significance of the new data shown in Figures 3e-f and Figure 5m-p. Complete replicate experiments would solidify this portion of the study. However, in lieu of performing complete replicates, the editors suggest that the authors may wish to soften their conclusions throughout (i.e. terms like "integral participants"–line 294-296). Rather, the authors might expand which aspects of the work are conclusive and which would fall under a proof-of-concept (line 469) category, citing the p-values of the data, and/or acknowledging the limitations of this part of the study and remaining unknowns about the role of antibodies in this model.***

We understand the concerns of the reviewer regarding scientific rigor for statistical analyses. We therefore asked for expert statistical analyses of Dr. Frank Klawon, one of the co-authors. The additional **Supplemental Tables 9 and 10** provide a transparent representation of the core data and statistical tests performed. In addition, we reformatted **Figure 3f** and **5m-p** to improve clarity. The statistical analyses are described in Methods **(lines 805-809)**:

P-values for all other comparisons were calculated using Welch’s t-test and the Wilcoxon-Mann-Whitney test. Significances are indicated and described in the figure legend. For calculation of differences between control mice and mice treated with mAbs (anti-CD20 or passive immunization), i.e. signal relative to baseline; we used the quotient of the medians.

The B cell depletion experiment was only requested by Reviewer 1 and it was a challenge for us to set it up as this kind of analyses is not commonly done for humanized mice. The data showed that B cell depletion after iDCgB immunization promoted a detectable HCMV/GLuc rebound **(P=0.051; Fig. 3f, Suppl Table 9B)**. Below is the revised text **(lines 299-310)**:

A clinical-grade anti-CD20 monoclonal antibody (rixathon, a rituximab biosimilar) was used in a pilot experiment to compare the effects of B cell depletion applied to HCMV-infected or to iDCgB-immunized and HCMV-infected mice. HCMV infection was monitored by optical imaging analyses at baseline before depletion and five days after B cell depletion (see experimental scheme and evidence of B cell depletion in **S 4b-d Fig;** see core data and statistics in **S9 Table**). B cell depletion promoted only a mild increase in HCMV/GLuc bioluminescence signal in non-immunized mice (n=3; P=0.63; 104% increase relative to baseline median values, **Fig. 3e-f, S9A Table**). For the iDCgB-immunized and then HCMV-infected mice, the effects of B cell depletion were associated with a more conspicuous HCMV rebound (n=4; P=0.05; 114% increase relative to baseline median values, **Fig. 3e-f, S9B Table**). Thus, these results indicated that the B cell responses elicited by iDCgB in this humanized model participated in the maintenance of HCMV immune control.

The passive immunization experiment was requested by all reviewers. Although the proof-of-concept experiment was performed with only 3 mice in each group (CTR Vs. mAbs) the results were significant in the context of passive immunization against infection (**P=0.0294, Fig. 5O, Suppl Table 10A**). Below is the revised text **(lines 365-388)**:

**Passive immunization with mAbs.** Ultimately, we evaluated in a proof-of-concept experiment if the best performing mAbs selected from the *in vitro* assays described above could provide *in vivo* protection against HCMV. In order to assess the effects of the mAbs on the HCMV infection, the antibodies DC06 and PR32 were administered i.v. (1 mg each) 16 weeks after HCT. For testing the effects against infection (“INFECT”, the mAbs were administered a day prior to HCMV/GLuc challenge and then weekly for three consecutive weeks (**Fig 5m;** see core data and statistics in **S10 Table**). At this time-point, optical imaging analyses were performed comparing non-treated HCMV-infected controls (CTR/HCMV; n=3) with mAb-treated mice (mAbs/HCMV n=3). Compared with the controls, mice receiving passive immunization showed significantly lower intensities of the bioluminescent signal (P=0.0294; 62.8% relative to median values obtained for controls) (**Fig 5n-o; S10A Table**). In a second step of the experiment, we evaluated if passive immunization could sustain an immune protection against HCMV reactivation when administered concurrently with G-CSF. At the endpoint of the experiment, two of the passively immunized mice sustained low levels of HCMV (average flux 1.9x10^3^ p/s) whereas one mouse showed viral rebound (flux 3.5x10^3^ p/s). Therefore, the passive immunization during HCMV reactivation yielded less pronounced and more variable results (P=0.348; 65.9% relative to median values obtained for controls; **Fig 5n-o; S10B Table**). Although not statistically significant (P=0.303) quantification of the viral copies in liver after reactivation showed that the passively immunized group retained only 14% of the PCR signal relative to the median values obtained for controls (**Fig 5p; S10C Table**). For analyses of BM, two out of three mice sustained low levels of viral copies but one mouse showed a HCMV rebound ( P=0.469; 32% reduction of median values obtained relative to controls; **Fig 5p; S10D Table)**. In conclusion, passive immunization with mAbs provided some level of protection against HCMV infection, but this protection was lessened upon HCMV reactivation.

Further, we softened our conclusions in several parts of the text:

Abstract **(lines 52-53):**

Passive immunization with these antibodies provided proof-of-concept evidence of protection against HCMV infection.

Author Summary **(lines 69-70):**

Proof-of-concept experiments indicated that administration of these monoclonal antibodies into mice protected them against HCMV infection.

Discussion **(lines 414-420):**

Further, B cell depletion studies designed to test the functional effects of B cells in the immune responses induced by iDCgB-immunization showed a modest viral rebound, indicating the relevance of the anti-gB humoral responses to maintain control against HCMV reactivations. A major limitation of this pilot study using a clinical anti-CD20 mAb was that it was performed with small cohorts of mice. Nonetheless, it provided an useful experimental approach to be further developed to address the possible clinical effects of anti-CD20 therapies in jeopardizing antiviral humoral responses.

We mentioned the limitations of the model **(lines 432-436)**:

The drawback of this *in vivo* HCMV model is the requirement of high levels of expertise and resources for generation of HIS mice and the fact that each experiment takes six months for completion. The conundrum is that HCMV infection is only consistently observed when applied after fifteen weeks after HCT, when human hematopoietic reconstitution in the mice is stable, robust and systemic.

**Part II – Major Issues: Key Experiments Required for Acceptance**

**Reviewer #1:**

***2.1. In the revised manuscript the authors provide additional experimental data that address all three of my original concerns.…Unfortunately, the new experiments (B cell depletion and adoptive antibody transfer) seem to have been performed only once with limited numbers of mice per experimental group. Therefore, statistical evaluation of the observed differences could not be performed. Both experiments should be at least repeated once and statistically evaluated.***

We apologize that we were not able to provide more replicate experiments for this review as additional experiments with larger cohorts would require 6 months to be completed. In addition, now with the coronavirus pandemic, we were asked not to start any additional animal experiments. Please see our response to the editors above (1.2).

**Reviewer #2:**

***2.2.The only remaining issue I have is regarding my question regarding the establishment of latency which, based on the authors response, may not have been made wholly clear. What isn't clear to me when reading the manuscript was whether the original establishment of latency was measured in the animals who were treated with control or the vaccine prior to infection. It stands to reason that if latency establishment is impaired by an anti-HCMV immune response then when reactivation analyses are performed there will be an effect seen but it may not necessarily be a block to reactivation but more reflect a difference on the initial impact on the seeding of latency in the model.***

The HCMV latency aspect and plans for a therapeutic vaccination model were addressed for the editors the above (see 1.1). For this current work, our focus was to evaluate the iDCgB vaccine in a protective setting shortly after HCT. In fact, we would like to mention a recent paper from our group that just came out in “Human Gene Therapy” exploring this humanized mouse model for testing of therapeutic gB-CAR-T cells in the setting of a pre-established HCMV infection. <https://www.liebertpub.com/doi/10.1089/hum.2019.149>.

***2.4. I appreciate it may not be easy to go back and assess latency if no tissue is available but I think it is worth noting in the discussion this limitation that the original establishment of latency may be impaired by the vaccine treatment unless there are data on latent load I have missed.***

Unfortunately we have not sacrificed mice to obtained tissues after infection. All experiments were terminated after reactivation promoted by G-CSF. Therefore, currently we would not be able to address these interesting questions, but thank you for the interesting suggestions.
