# Supplementary material for: Repertoire characterization and validation of gB-specific human IgGs directly cloned from humanized mice vaccinated with dendritic cells and protected against HCMV
Source: PLoS Pathog. 2020 Jul 15;16(7):e1008560. doi: 10.1371/journal.ppat.1008560 (PMC7363084; doi:10.1371/journal.ppat.1008560)
Supplement: Attachment — Submitted filename: Theobald R3 PlosPathogens Rebuttal.doc [file ppat.1008560.s019.doc]

MHH - Hämatologie, Hämostaseologie und Onkologie - 30623 Hannover

**Zentrum Innere Medizin**

**Hämatologie, Hämostaseologie und Onkologie**

**Und Stammzelltransplantation**

**Prof. Dr. med. Arnold Ganser, Direktor**

Regenerative Immune Therapies Applied

Prof. Dr. Renata Stripecke

Telefon:	++49 (0) 511 532-6999

Fax:	++49 (0) 511 532-6975

[Stripecke.renata@mh-hannover.de](mailto:Stripecke.renata@mh-hannover.de)

www.stripecke-mhh.de

Carl-Neuberg-Straße 1 OE6860

30625 Hannover

Telefon: 0511 532-0

www.mh-hannover.de

**Christopher M. Snyder (Guest Editor)**

**Blossom Damania (Section Editor)**

**Kasturi Haldar (Editor-in-Chief)**

**Michael Malin (Editor-in-Chief)**

**PlosPathogens**

**Ref: PPATHOGENS-D-19-01985-R3**

**30. November 2026**

Dear Dr. Haldar, Dr. Malin, Dr. Damania and Dr. Snyder,

Thank you very much for your expedited re-revision and detailed attention to our manuscript: “*Repertoire characterization and validation of gB-specific human IgGs directly cloned from humanized mice vaccinated with dendritic cells and protected against HCMV”.*

We truly apologize that there were still some pending clarifications. The focus of this work was primarily on analyzing the vaccination effects on primary infection. Therefore, we revised the text to soften the statements regarding the effects of vaccinations on the latent viral load reactivations.

The revisions were performed in:

1. Results,
2. Discussion, and
3. Throughout the text

Our revisions were provided point-by-point below and they are highlighted in the text for this current review in grey (previously for reviewer 1 in yellow and for reviewer 2 in green). A clean revised version was also uploaded.

If you still have recommendations for different phrasing styles, please do not hesitate to contact me.

Sincerely yours,

**Prof. Dr. Renata Stripecke**

**Department of Hematology, Hemostasis,**

**Oncology, and Stem Cell Transplantation**

**Hannover Medical School, Germany**

**Part I – Summary**

**Editor:**

***I think there is still a mis-understanding about the clarification requested by Reviewer #2. This reviewer is referring to this prophylactic vaccination setting described in this study, and asking whether the iDC vaccinations conducted between weeks 6 and 10, affect the total HCMV latent loads established after the week 17 HCMV infection, but prior to the reactivation started at week 24. In other words, just before the G-CSF treatment in week 24, do all of the vaccinated and unvaccinated mice have equivalent amounts of viral DNA? For example, if the vaccinated mice controlled the primary HCMV infection in week 17 better, it could result in reduced latent viral DNA by week 24, which could then lead to reduced luciferase signal after reactivation (e.g. in Figure 2d).***

The effects of iDCgB on the HCMV latent loads is a valid question that remains to be clarified in the future, with more complex and demanding models (with longitudinal and cross-sectional analyses) and much larger cohorts of humanized mice. For the current studies we did not perform cross-sectional or longitudinal optical imaging or qPCR analyses to evaluate if the iDCgB vaccine could influence HCMV latency or reactivation. The iDCgB vaccine was tested primarily to evaluate the prophylactic effects in reducing primary infection. The administration of G-CSF was performed in order to amplify the sensitivity for improving the detection of residual latent HCMV infections. The amendments and revisions in the text can be found in the sections mentioned below.

**Introduction (lines 118-124):**

Other noteworthy HCMV infection models have been reported (11), but the advantage of our model is the relatively simple non-invasive monitoring of the HCMV infection and the possibility of highly sensitive amplification of the viral detection after G-CSF treatment in order to predict if a vaccine or therapy can control or mitigate a primary viral infection. Thus, this model is useful for testing the protective effects of human-specific vaccines or immunotherapies and to evaluate the distinct immunological mechanisms that block or reduce a HCMV primary infection.

**Results (lines 208-241):**

Here, this model was used to evaluate primarily the prophylactic effects of iDCgB vaccination against a HCMV primary infection. If protection was not complete and a latent viral reservoir was eventually established, the virus could be reactivated with a G-CSF stimulus to induce viral outburst to facilitate monitoring. Humanized mice were pre-immunized with iDCgB (n=17; 3 different CB donors; immunizations were administered at weeks 6, 7, 10 and 11 after HCT). Non-immunized mice were included as controls (n=12). Seventeen weeks after HCT, all mice were challenged with HCMV-GLuc to produce a primary infection (**Fig 2a**). From week twenty four to twenty five after HCT, once viral latent infection was established, mice were then treated daily with G-CSF to stimulate HCMV/GLuc reactivation in order to amplify a putative residual viral infection in mice, making the monitoring more sensitive and traceable (**Fig 2a**). In order to minimize the handling of the mice (which could potentially interfere with the experimental outcome), the analyses of the immunological responses and viral load were performed only at the experimental endpoint, one week after G-CSF reactivation. Both immunized and non-immunized cohorts showed similar stable long-term huCD45 and huCD3 immune reconstitution in blood (**Fig 2b**), but iDCgB-vaccinated mice showed higher numbers of detectable LN structures, indicating a typical immunization effect (**Fig 2c**). Bioluminescence signal was conspicuous in anatomical regions of liver (LI) and salivary glands of all HCMV-infected control mice but barely noticeable for 13/17 iDCgB-immunized mice (**Fig 2d**). The highly sensitive monitoring of the full-body bioluminescence signal indicated that a residual HCMV infection was still measurable in the iDCgB-immunized mice but the signals were significantly decreased compared with control mice (**Fig 2e**). HCMV viral loads in mice were quantified by qPCR analyses of genomic viral copies in DNA extracted from LI and BM. Blood was not analyzed, because in previous work we observed that qPCR analyses of blood were not quantitatively reliable, with a high frequency of false-negative results (16). Significantly decreased levels of HCMV DNA load were measured in 93% of iDCgB-immunized mice (**Fig 2f**, **S2 Table**). HCMV was undetectable for 9/16 of the analyzed immunized mice for which DNA was available, in these cases indicating a full protection. These results showed that prophylactic iDCgB immunization efficiently minimized or even fully protected HIS mice against a primary HCMV infection. A residual HCMV load and spread could still be detectable in some of the vaccinated mice, with the caveat that G-CSF was purposely administered in order to amplify a putative residual latent viral reservoir and to make the readout of this model highly sensitive. Whether the prophylactic iDCgB vaccine could promote immunologic effects to mitigate reactivation of the latent reservoir *per se* remains to be evaluated with a different experimental design, i.e. with cross-sectional or longitudinal analysis.

***Thus, the vaccination might be having its greatest effect on the primary infection and the establishment of latency after week 17, rather than on the reactivation from latency after week 24.***

Correct, this is the valid conclusion in the strict sense. The discussion was revised and amended.

**Discussion (lines 427- 442):**

The prophylactic iDCgB vaccination in the model resulted in a full protection against HCMV primary infection in the majority of the HIS mice. In the strict sense, the effects of iDCgB immunization to mitigate latency and/or to prevent HCMV reactivation remain to be further evaluated in longitudinal or cross-sectional experimental models. In the broad sense, iDCgB administered prior to a HCMV primary infection, showed marked effects in the development IgG and IgA class-switched B cells. Bioinformatics analyses of large datasets predicted IgG, IgA and CD4^+^ T cell as key biomarkers of response accompanying iDCgB immunization. Interestingly, these markers have also been associated with protection against HCMV in humans in keeping the infection dormant (38, 39). Further, B cell depletion studies designed to test the functional effects of B cells in the immune responses induced by iDCgB-immunization showed a modest viral rebound, indicating a possible relevance of the anti-gB humoral responses to maintain control against reactivation of latent HCMV reservoir. A major limitation of this pilot study using a clinical anti-CD20 mAb was that it was performed with small cohorts of mice. Nonetheless, it provided a useful experimental approach to be further developed to address the possible clinical effects of anti-CD20 therapies in jeopardizing the immune control of the silent HCMV latency, which could then progress towards viral reactivation.

**Discussion (lines 458- 478):**

…Therefore, more complex longitudinal or cross-sectional analysis of HIS mice are very demanding as several HCTs have to be established in parallel in order to allow statistical power of analyses.

For the current studies, we opted to evaluate the effects of prophylactic vaccination with iDCgB prior to a HCMV primary infection, because we were primarily interested in the early immune reconstitution and antiviral responses shortly after HCT. Further, a potent immunization against a primary infection could help to mitigate the establishment of a HCMV latent reservoir. However, from the clinical perspective, the highest risk group is when the HCT donor is HCMV-seronegative and the recipient is seropositive. In this case, the latent viral reservoir of the recipient is intrinsic. In this more complex scenario, the pre-existing latent viral reservoirs can disseminate during the early stages of immune reconstitution, when the recipient is immune compromised, causing serious HCMV disease. Therefore, there is an important need and a large interest to develop novel immunotherapeutic strategies to eradicate HCMV latent infections or to prevent the latent infection to reactivate (recently reviewed in ref. (44)). Since during latency the viral gene expression is largely suppressed, indeed it would more challenging to expect immune responses against latency-associated genes (44). Remarkably, cytotoxic CD4^+^ T cells producing IL-10 and directed against the latency-associated proteins LUNA and UL138 have been detectable in healthy HCMV-positive donors (45). However, these T cell responses directed to latency-associated antigens seem to be skewed to become immunosuppressive, and this is an immune escape mechanism that could potentially sustain viral latency.

**Discussion (lines 498- 504):**

Thus, the promising prophylactic results obtained with the iDCgB vaccine in this HIS/ HCMV model could be translated clinically to the solid organ transplantation setting, when HCMV seronegative patients are on the waiting list and very likely will be transplanted in a near future with organs from seropositive donors. The data also warrant the testing iDCgB in the therapeutic setting, i.e. to predict if high-risk HCMV seropositive HCT patients transplanted with stem cells from seronegative donors (including cord blood donors) could be protected.

Further, other minor corrections were performed and are indicated in the text in grey to avoid ambiguities regarding HCMV infection versus latency and reactivation.
